# Operando UV/vis Absorption Spectroscopy for Studying the Nitrate to Ammonia Conversion on Cu_2_O‑Based Electrodes

**DOI:** 10.1021/acscatal.5c07326

**Published:** 2026-01-16

**Authors:** Maria Huidobro, Luis Romay, Martin Perez-Estebanez, Aranzazu Heras, Juan V. Perales-Rondon, Alvaro Colina

**Affiliations:** † Department of Chemistry, 16725 Universidad de Burgos, Pza. Misael Bañuelos s/n, Burgos E-09001, Spain; ‡ Hydrogen and Power-to-X Department, Iberian Centre for Research in Energy Storage, Polígono 13, Parcela 31, ≪El Cuartillo≫, Cáceres E-10004, Spain

**Keywords:** nitrate, electrocatalysis, operando spectroscopy, electrochemistry, UV/vis absorption spectroelectrochemistry, ammonia

## Abstract

The electrochemical conversion of nitrate to ammonia was proposed as a feasible strategy for ammonia production. However, a deep understanding of the reaction mechanism and catalyst transformation is still needed to reach real applications. Herein, operando 2D-UV/vis absorption spectroelectrochemistry (2D-UV/vis SEC) was used to study in detail the reaction dynamics, as well as the transformation of the catalyst for the nitrate reduction to ammonia on Cu_2_O-based electrodes. 2D-UV/vis SEC combines two simultaneous UV/vis absorption spectroelectrochemistry measurements to obtain information on a given system from two different points of view: normal and parallel configurations. The UV/vis signal in the parallel arrangement facilitates tracking of the reduction of nitrate to nitrite, providing kinetic information about the process. Conversely, the UV/vis signal in the normal configuration reveals the transformation of the catalyst, providing more detailed information about the changes occurring on the electrode surface during the reaction. Spectroelectrochemistry responses have shed more light on the kinetics of the process under the operating conditions. The results show that at the selected potential window for nitrate reduction, the Cu_2_O surface is mostly reduced to Cu^0^, as evidenced by the changes observed in absorbance in the normal configuration. In addition, experiments performed at different initial nitrate concentrations reveal that nitrite reduction is hampered by the nitrate concentration, demonstrating the key role of the nitrate concentration in the reaction pathway. This work highlights the usefulness of 2D-UV/vis SEC in untangling the complex dynamics of the reaction intermediates and products as well as the transformation of the catalyst surface in electrocatalytic processes.

## Graphical abstract

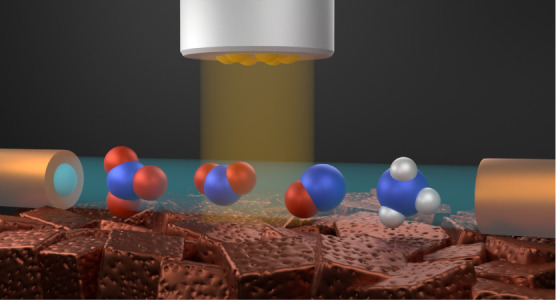

## 1 Introduction

Ammonia is a widely used chemical compound which plays a fundamental role in our society.^1^ It can be used as a precursor for fertilizers, as a raw material for chemical, fuel, or pharmaceutical production, and as an energy vector due to its high energy density.^2–5^ Currently, the supply of ammonia is closely linked to the Haber–Bosch process (HB), which involves a thermocatalytic reaction between hydrogen and nitrogen to produce ammonia. However, HB is an energy-intensive process that requires high temperatures and pressures,^3^ relying on fossil fuels and contributing significantly to CO_2_ emissions which account for at least 1.2% of global anthropogenic emissions.^6^ Therefore, new routes for ammonia production powered by renewable sources are being intensively explored.^7,8^ Concurrently, an increase in nitrate concentrations in aquifers has been identified, linked to the intensive use of fertilizers and industrial waste, posing a threat to aquatic ecosystems, animals, and human health. Thus, there is a growing interest in sustainable techniques for eliminating nitrate contaminants, aiming to prevent the disruption of the nitrogen cycle.

Considering these problems, electrochemistry has emerged as an ideal tool for the development of methodologies to produce ammonia while removing excess of nitrate from contaminated water, rebalancing the nitrogen cycle.^9,10^ Electrochemical nitrate reduction to ammonia (NRA) is a nonspontaneous reaction which requires the application of a voltage and the use of an appropriate electrocatalyst. In alkaline media, NRA is complex and involves the transfer of 8 electrons and 9 hydroxyl anions (eq 1). The number of reaction steps increases the possibility of obtaining stable intermediates, making their study challenging. To better understand the reaction mechanism and to control the process for future large-scale applications, monitoring the reaction intermediates as well as the transformations occurring on the catalyst surface is of the utmost importance.

1 $$NO_{3}^{-}\left(aq\right)+6H_{2}O+8e^{-}\to NH_{3}\left(aq\right)+9OH^{-}$$

Among the techniques utilized to monitor reaction mechanisms, Raman spectroscopy, FTIR spectroscopy, or X-ray absorption spectroscopy (XAS) are the most commonly used.^11,12^ Raman spectroscopy provides accurate vibrational information on both the reaction intermediates and products as well as on the surface structure of the catalyst. However, the major drawback of this technique is its lack of sensitivity, which is reflected in the low intensity of the Raman bands. To address this issue, it is imperative to amplify the Raman signal using a surface-enhanced Raman scattering (SERS) strategy, which poses a major challenge. FTIR spectroscopy also provides information about the vibrational modes of molecules on the electrochemical interface, but in this case, the use of aqueous solutions is challenging because of the interference of solvent bands in the recorded signal. On the other hand, XAS is a powerful technique for the study of the surface structure of the catalyst, as well as the reconstruction phenomena on the electrode surface, since it allows the determination of the oxidation state or the coordination number of the catalyst metal. However, it requires expensive instruments and specialized facilities, which hinders its widespread application.^13^

Operando techniques, such as UV/vis absorption spectroelectrochemistry (UV/vis-SEC), are surely promising tools for providing complementary information about reagent and intermediate dynamics during the electrode process. UV/vis-SEC is an operando technique that has undergone significant development in recent years^14,15^ and has proven to be useful for both qualitative and quantitative analyses. UV/vis-SEC can be performed in different configurations to sample the working electrode surface and diffusion layer, enhancing the versatility of the technique. The combination of multiple configurations helps to deconvolve the signals and easily identify processes taking place on the electrode surface and at the diffusion layer.

The most frequently used optical arrangements in the literature are the parallel and normal configurations, which can be performed simultaneously in the so-called operando 2D-UV/vis absorption spectroelectrochemistry (2D-UV/vis SEC).^15,16^ In the parallel configuration, the light beam samples the solution adjacent to the electrode surface, tracking the evolution of the species in the diffusion layer. In the normal configuration, the light beam is focused perpendicular to the working electrode, making it possible to obtain information about both the electrode surface and the solution adjacent to it; therefore, it could be useful to follow the evolution of the species in the diffusion layer, the species absorbed on the electrode surface, and the changes occurring on the catalyst surface. The combination of these two configurations in a single experiment provides correlated information and a more complete description of the NRA dynamics.

In this study, 2D-UV/vis SEC^14^ has been used to study the NRA by evaluating the evolution of the species of interest in solution, such as nitrate and nitrite, in the parallel configuration and monitoring the changes that occur on the electrode surface in the normal configuration. In addition to that, 2D-UV/vis SEC has been utilized to evaluate the dynamics of the process at different initial concentrations of nitrate, providing additional insights into the kinetics of the whole reaction process.

## 2 Materials and Methods

### 2.1 Reagents and Materials

Potassium chloride (KCl, >99%, Acros Organics), potassium hydroxide (KOH, EMSURE ACS, Sigma-Aldrich), potassium nitrate (KNO_3_, >99%, Sigma-Aldrich), sodium nitrite (NaNO_2_, >97%, Sigma-Aldrich), and sodium hydrogen carbonate (NaHCO_3_, 99.7%, Panreac) were used as received, without further purification. All solutions were prepared using ultrapure water obtained from a Millipore DirectQ purification system (Millipore, 18.2 MΩ cm resistivity at 25 °C).

### 2.2 Instrumentation

2D-UV/vis SEC experiments were performed using two customized SPELEC (Metrohm-DropSens), controlled by DropView SPELEC software (Metrohm-DropSens); each SPELEC includes a light source, a potentiostat, and a spectrophotometer. A reflection probe (DRP-RPROBE-VIS-UV, Metrohm-DropSens) was used to perform SEC measurements in the normal configuration. Two 100 μm bare optical fibers (Avantes) were used to perform measurements in the parallel configuration.

A three-electrode system was used to perform all experiments. A copper disk (3.18 mm, 99.999%, Alfa Aesar) was used as the working electrode, a reversible hydrogen electrode (RHE) or a commercial Ag/AgCl was used as the reference electrode, and a platinum wire was used as the counter electrode. Unless otherwise stated, all potentials were referred to the RHE.

A Zeiss GeminiSEM560 field-emission scanning electron microscope (FE-SEM) was used to obtain the SEM images of the WE surface using an electron beam of 3 kV. This FE-SEM was equiped withan EDX analyzer, which was used to carry out EDX mapping. An electron beam of 3 kV was used for EDX mapping.

X-ray Diffraction (XRD) measurements were performed using a D8 Discover A25 (Bruker) X-ray diffractometer, using a Cu Kα radiation source (λ = 0.154 nm). Scans were recorded in the range of 2θ = 25°–80°.

### 2.3 2D-UV/vis Absorption Spectroelectrochemical Cell

A new SEC cell was developed to perform the measurements of the NRA with 2D-UV/vis SEC using a copper disk as the working electrode. Figure 1A shows the schematic of the 2D-UV/vis SEC cell used in these experiments. Briefly, it consists of two Teflon pieces (2, 5) with magnets in each corner to hold a thin *Viton* layer (3) to prevent leakage of the solution. The copper working electrode (6) is embedded in the bottom Teflon piece (5), placed under the reflection probe (1), which provides the optical response in the normal arrangement. This reflection probe consists of 6 optical fibers illuminating the sample and a central optical fiber which collects the reflected light beam. Two 100 μm optical fibers (4) are aligned, maximizing the light passing through them, and attached to the piece at the bottom (5) to obtain the parallel response, that is, parallel to the working electrode surface. The distance between the two optical fibers is measured, and the values ranged from 1.16 to 1.60 mm. Romay et al.^17^ provided an excellent description of the positioning of the two optical fibers relative to the electrode surface in a similar cell. In summary, the core of the optical fiber is coated with a cladding layer with a thickness of 50 μm, which means that the diffusion layer is sampled 50 μm above the electrode surface. Given that the diameter of the optical fibers is 100 μm, only the area of the diffusion layer determined by the cross section of the fiber is interrogated. Importantly, the optical response is influenced by the scan rate, achieving a steady state at very low scan rates because the reaction products can diffuse away from the sampled zone.^15^ The cell is designed with a specific shape to fix the optical fibers close to the working electrode and to place the reference (7) and counter (8) electrodes separated from the working electrode (6), which is placed in a different quasi-compartment, as depicted in Figure 1.

**Figure 1:**
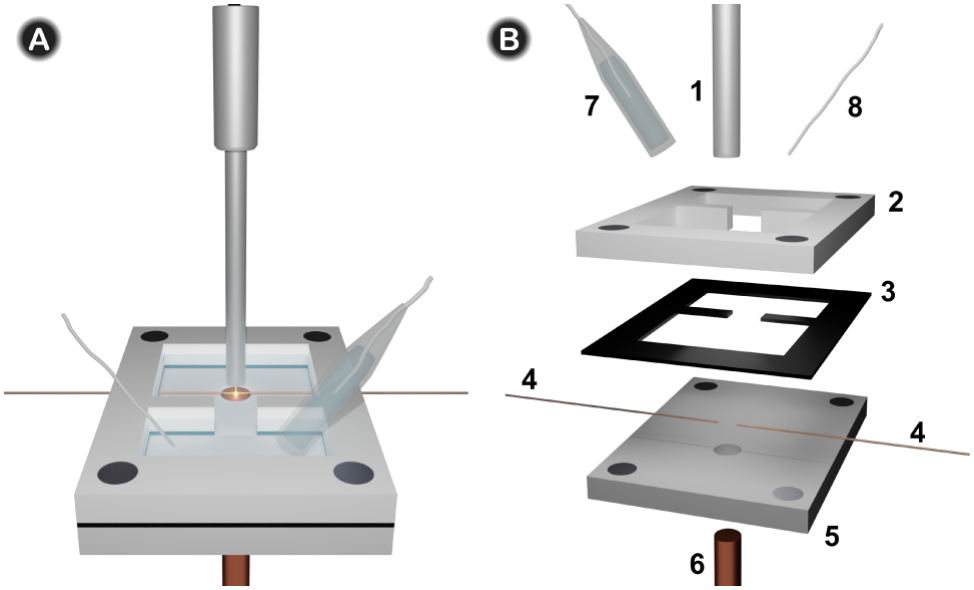
(A) Schematic of the 2D-UV/vis SEC cell utilized in this study. (B) Detailed schematic of the cell: (1) reflection probe, (2, 5) Teflon pieces, (3) *Viton* layer, (4) illumination and collection optical fibers in the parallel configuration, (6) copper disk used as the working electrode, (7) reference electrode, and (8) counter electrode.

### 2.4 Synthesis of Cu_2_O Catalyst

Cu_2_O electrocatalyst was prepared in two stages following a protocol described in the literature:^18^ an electrochemical modification followed by a chemical treatment of a Cu disk. Before carrying out the modification, the Cu disk electrode was thoroughly polished with 0.05 μm alumina to obtain a mirror-like finish, and it was sonicated in deionized water to clean its surface. The first stage consisted of an electrochemical oxidation of the Cu electrode by applying +1.10 V vs Ag/AgCl in 0.1 M KCl for 100 s.^18^ Subsequently, the electrode was rinsed with deionized water and subjected to a second stage. This consisted of a chemical treatment by immersing the electrode in a solution of 0.1 M NaHCO_3_ for 10 min. After this step, a Cu_2_O-based electrode composed of superficial nanocubes was obtained and used for the NRA.^18,19^

### 2.5 Electrochemical Nitrate Reduction

Once the working electrode surface was modified, electrochemical reduction of nitrate to ammonia experiments were carried out in a solution of KNO_3_ in 1 M KOH. Linear sweep voltammetry (LSV) was performed from +0.40 V to −0.60 V; however, SEC only provides information between +0.40 V and −0.40 V since bubble production hinders the spectroscopic tracking of the evolution of the reaction under study. A scan rate of 0.02 V s^–1^ was set for all of the experiments. The integration time was 10 ms in the parallel configuration, averaging 5 spectra, and 100 ms in the normal configuration. The experiments were conducted under atmospheric conditions without implementing a deoxygenation step. This approach was adopted to investigate the mechanistic aspects of the nitrate reduction reaction under conditions that more closely resemble real-world applications, where a deoxygenation stage is not necessary.

Nevertheless, the SEC cell is also suitable for experiments conducted under an inert atmosphere. Deoxygenation with argon is preferable as it allows an argon layer to be created over the solution contained in the SEC cell, which limits the entry of oxygen into the solution through its surface.

## 3 Results and Discussion

### 3.1 2D-UV/vis SEC of NRA on Cu_2_O-Based Electrodes

NRA experiments were performed for the Cu_2_O-based electrode in an electrolytic medium of 0.1 M KNO_3_ in 1 M KOH. 2D-UV/vis SEC experiments with LSV were performed with and without KNO_3_ in 1 M KOH, and the evolution of absorption spectra in the normal and parallel configurations was recorded simultaneously.

The reference spectrum in the two optical configurations was taken just before starting the experiment; therefore, changes in the absorbance were measured. Consequently, a positive absorbance indicates the generation of a product of the reaction, whereas a negative absorbance indicates the consumption of reactants. In the parallel arrangement, the optical fibers interrogate the solution adjacent to the working electrode, which means that only information related to the spectral changes occurring in the first 100 μm of the diffusion layer is obtained. In addition, changes in absorbance in the normal arrangement (which involve working in the reflection mode) that are not visible in the parallel configuration could be associated with the modification of the catalyst during the electrochemical experiment. Particularly in the normal arrangement, the reference spectrum is the one obtained with the catalyst formed on the electrode. Thus, changes of absorbance with respect to this initial spectrum are more representative than absolute absorbances in reflection mode because of the high change of the complex index of refraction of the material deposited on the electrode with respect to the one of the copper electrodes. For simplicity, we first analyze the results obtained for the parallel arrangement. Figure 2 summarizes the 2D-UV/vis SEC experiments for the NRA with (solid lines) and without KNO_3_ (dashed lines), where only the results in the parallel configuration are shown. Figure 2A shows the evolution of the absorption spectra during the LSV where two distinct absorption bands emerged during the experiment. The band centered at 350 nm exhibits positive absorbance values, whereas the other band, centered at 300 nm, displays negative absorbance values. From previous studies, nitrite has been proposed as one of the main stable intermediates in the NRA mechanism,^3,20,21^ so further exploration of both nitrate and nitrite absorption behavior was performed in this study. Figure 2C shows the UV/vis absorption spectra of a 0.1 M KNO_3_ solution and a 0.1 M NaNO_2_ solution, both in 1 M KOH. As can be clearly observed, the positions of the characteristic absorption bands of these two anions coincide with the bands observed during the 2D-UV/vis SEC experiment. Therefore, the band at 350 nm is related to nitrite evolution, and the band at 300 nm is ascribed to nitrate consumption, as shown in Figure 2A and Figure S1.

**Figure 2:**
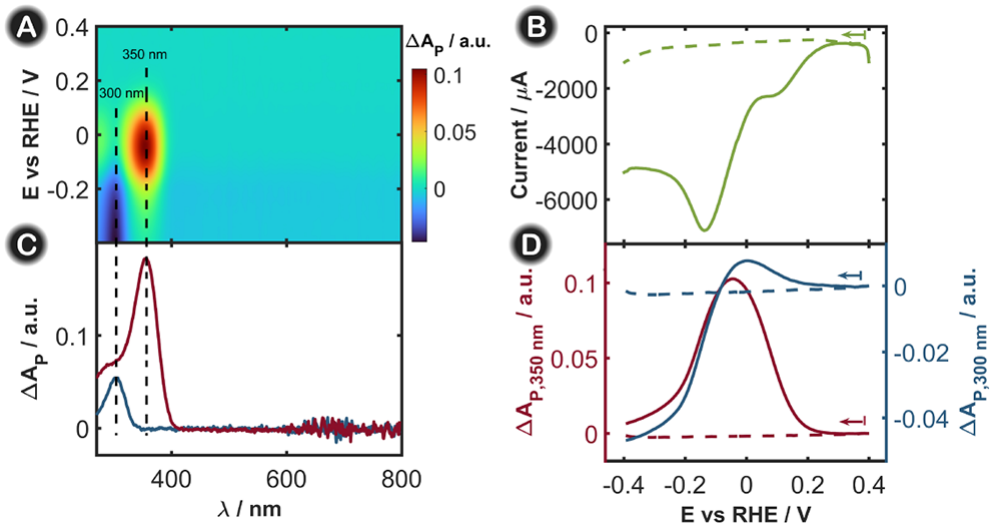
(A) Contour plot showing the evolution of the absorption spectra in the parallel configuration during the LSV of a 0.1 M KNO_3_ solution in 1 M KOH. (B) LSV of a 1 M KOH solution in the absence of KNO_3_ (green dashed line) and with 0.1 M KNO_3_ (green solid line). (C) UV/vis absorption spectra of 0.1 M KNO_3_ (blue line) and 0.1 M NaNO_2_ (red line), both in 1 M KOH. (D) LVAs in the parallel configuration at 350 (red line) and 300 nm (blue line). Experiments were conducted in the absence (dashed lines) and presence (solid lines) of 0.1 M KNO_3_. The scan rate was fixed at 0.02 V s^–1^, and the integration time was 10 ms.

The LSV recorded in the presence of KNO_3_ (Figure 2B, green solid line) shows two main reduction peaks, at +0.10 V and at −0.14 V. According to the literature, the first cathodic peak centered at +0.10 V is attributed to the nitrate reduction to generate nitrite, which is attributed to the maximum nitrite production during NRA.^18,22^ In addition, the reduction peak at −0.14 V accounts for the further reduction of NO_2_^–^ to products with a lower oxidation state of nitrogen, as has been stated in the literature and considering similarities in the curves for the NO_3_^–^- and NO_2_^–^-containing electrolytes.^5^ To confirm the assignment of the voltammetric peaks, SEC experiments with 0.1 M KNO_2_ were performed, Figure S2. As can be seen in the corresponding voltammogram, Figure S2A, the peak at +0.10 V vanishes, suggesting that this first reduction peak is mainly related to the formation of nitrite from the reduction of nitrate. It is important to mention that, compared to a typical pure electrochemical experiment, the potential window shown in this experiment was shorter because bubble production at more cathodic potentials interferes with the optical measurements, hindering the collection of reliable spectra. These bubbles can be assigned to the generation of NH_3_ when the solution is saturated (or even some amount of H_2_), as has been previously demonstrated in the literature.^23^ Therefore, all SEC measurements were plotted up to −0.40 V in the cathodic direction. The same experiment was performed in the absence of nitrate in the solution (Figure 2B, green dashed line), where no reduction peaks were observed, as expected from previous results obtained with Cu_2_O electrodes in alkaline media.^18^ Plotting the evolution of the absorbance in parallel configuration at a specific wavelength as a function of the applied potential (linear voltabsorptogram, LVA) provides a better understanding of the UV/vis absorption changes during the LSV. Figure 2D shows the LVAs at 300 and 350 nm, which correspond to the nitrate and nitrite absorption bands, respectively. As can be observed for nitrite (Figure 2D, red solid curve), the signal starts to increase at approximately +0.20 V, reaching a maximum at −0.05 V. Since nitrite was not present in the initial solution, these changes indicate that nitrite is initially electrogenerated when the cathodic process begins and starts disappearing thereafter (from −0.10 V to more negative values) to form other reaction intermediates. Therefore, nitrite is an intermediate of NRA, participating in an important step of this reaction, as will be shown below. On the other hand, for nitrate dynamics (Figure 2D, blue solid curve), we should expect a continuous decrease in absorbance, as nitrate is consumed at the electrode surface as the experiment proceeds. However, there is a slight increase in absorbance from +0.20 to 0.00 V, at which point the absorbance dropped to negative values, continuously decreasing in the cathodic region, indicating the depletion of nitrate in the diffusion layer. The increase in nitrate absorbance at +0.20 V is related to the overlap of the nitrate and nitrite absorption bands. As nitrite has a higher molar absorptivity coefficient^24–26^ and both absorption bands partially overlap (Figure 2C), when nitrite starts to evolve, its signal is more pronounced than that corresponding to nitrate depletion, dominating the entire spectroscopic behavior. Nevertheless, as the experiment proceeds, the changes in nitrate concentration (consumption) are greater, and nitrite starts to be transformed in the next step of the reaction. Therefore, the nitrate spectrum dominates the overall behavior of the SEC experiment. Blank experiments in the absence of nitrate were performed, showing that the absorbance does not change during the voltammetric experiment (Figure 2D, dashed lines) either at 350 or 300 nm.

With the aim of confirming the identity of voltammetric signals, additional SEC experiments for nitrate reduction (0.1 M KNO_2_) were also carried out in the same electrolytic conditions. The voltabsorptogram at 350 nm shows that the onset potential for nitrite reduction is located at more negative values than +0.10 V (Figure S2A and C). By comparison of the LVAs of the solution containing nitrate with that containing only nitrite, the assignment of the reduction peaks in the LSV is demonstrated more clearly, Figure S2C. Moreover, experiments starting with NaNO_2_ were performed, showing very similar results, ruling out any effect of the cation in the reduction process (Figure S2B and D).

The data obtained from the 2D-UV/vis SEC experiments in the normal arrangement are remarkably similar to those obtained in the parallel configuration in the UV region of the spectra. However, notable differences were observed in the visible region of the absorption spectra. Figure S3 summarizes the SEC experiment in the normal configuration for the NRA of a 0.1 M KNO_3_ solution in 1 M KOH. The trend of the spectra recorded in the normal configuration along the LSV is shown in Figure S3A. In the UV spectral region, the two bands centered at 300 and 350 nm showed the same behavior as that shown in the parallel configuration (compare Figure 2D and Figure S3B). It should be noted that the signals corresponding to species in solution have lower intensity since the optical pathway is smaller in the normal configuration (thickness of the diffusion layer, in the order of hundreds of microns) than in the parallel configuration (between 1.16 and 1.60 mm). As mentioned above, one advantage of the normal configuration is that it is easy to track information related to the structural changes occurring on the working electrode surface. Indeed, 2D-UV/vis SEC allows us to distinguish between processes taking place in solution, which are more accurately described in a parallel configuration, and processes taking place on the electrode surface, which are observed only in the normal configuration. As can be seen in Figure S3A, in the visible spectral region, there is a broad band centered at 650 nm, which is not observed in the parallel configuration (Figure 2A). The evolution of the LVA at 650 nm shows that the absorbance decreases linearly with the applied potential between +0.40 and −0.40 V (Figure S3C), without a clear change in the absorbance related to an electrochemical reaction. Moreover, the small change in absorbance could be related to changes in the reflectivity of the electrode induced by the applied potential since a deep change in the structure of the catalyst promotes a clear change in the observed spectra, as will be shown in the next section.

### 3.2 2D-UV/vis SEC of Cu_2_O-Based Electrodes in 1 M KOH

It is important to note that before performing the NRA experiment (Figures 2 and Figure S3), four consecutive reduction scans were carried out in 1 M KOH with the electrode freshly prepared, changing the KOH solution and leaving the electrode at open circuit potential at the end of each scan (same experimental parameters as those utilized in Figure 2). The aim of performing these reduction scans was, on the one hand, to ensure that the experiments always started with a similar surface in terms of its oxidation state and, on the other hand, to prevent cathodic currents from being influenced by processes originating from the electrode surface itself, such as metal dissolution, catalyst transformation, etc. In several studies found in the literature, before the NRA test, LSV curves are performed until the polarization curves achieve a steady-state behavior.^5,27^ In this section, the changes in the catalyst during this pretreatment will be tracked by using the normal configuration. It should be noted that negligible absorbance changes were observed in the parallel configuration.

Before the experiment was performed, the freshly prepared electrode was thoroughly characterized to confirm the identity of the oxide material. Figure S4 shows the XRD and SEM/EDX of the electrode before cycling in the blank, confirming that nanocubes on the electrode surface are mostly composed of Cu_2_O. After 4 subsequent LSVs performed on the same electrode in a solution containing only 1 M KOH, a stable and reproducible electrode surface was obtained. The first LSV of the catalyst in 1 M KOH (Figure S5A, blue curve) presents the greatest change in terms of current compared with the subsequent LSVs (Figure S5A). This is an expected result since during the first LSV, surface oxides can be reduced,^28^ causing the electrode to undergo the greatest changes in terms of cathodic currents, which can be associated with changes in the optical response. In subsequent LSVs, the electrochemical and optical responses were more reproducible and stable. Figure 3 shows the results obtained with a Cu_2_O electrode in 1 M KOH during the first two LSVs of a 2D-UV/vis SEC experiment in the normal configuration. The evolution of the spectra during the first two LSVs is clearly different (Figure 3A and C, Figure S5B and C). During the first reduction scan, the absorbance changes from −0.2 to 0.4 au (Figure 3A and Figure S5B), whereas in the second reduction scan, the absorbance changes only from 0 to −0.1 au (Figure 3C and Figure S5C). In the first reduction scan (Figures 3A and Figure S5B), a negative absorption band around 400 nm decreased, and a broad absorption band around 650 nm increased from −0.20 V to −0.40 V, which is related to the reduction of Cu_2_O nanocubes^29–31^ to Cu^0^ (Figure S5G and Figure S6), as described in the literature from synchrotron measurements.^32^ As was aforementioned, negative absorption bands in UV/vis-SEC are related to the consumption of reagents present in the initial state of the experiment. Therefore, the band around 400 nm can be ascribed to Cu_2_O, which is being reduced to form Cu^0^ nanoparticles, which exhibit a positive absorption band around 650 nm.

**Figure 3:**
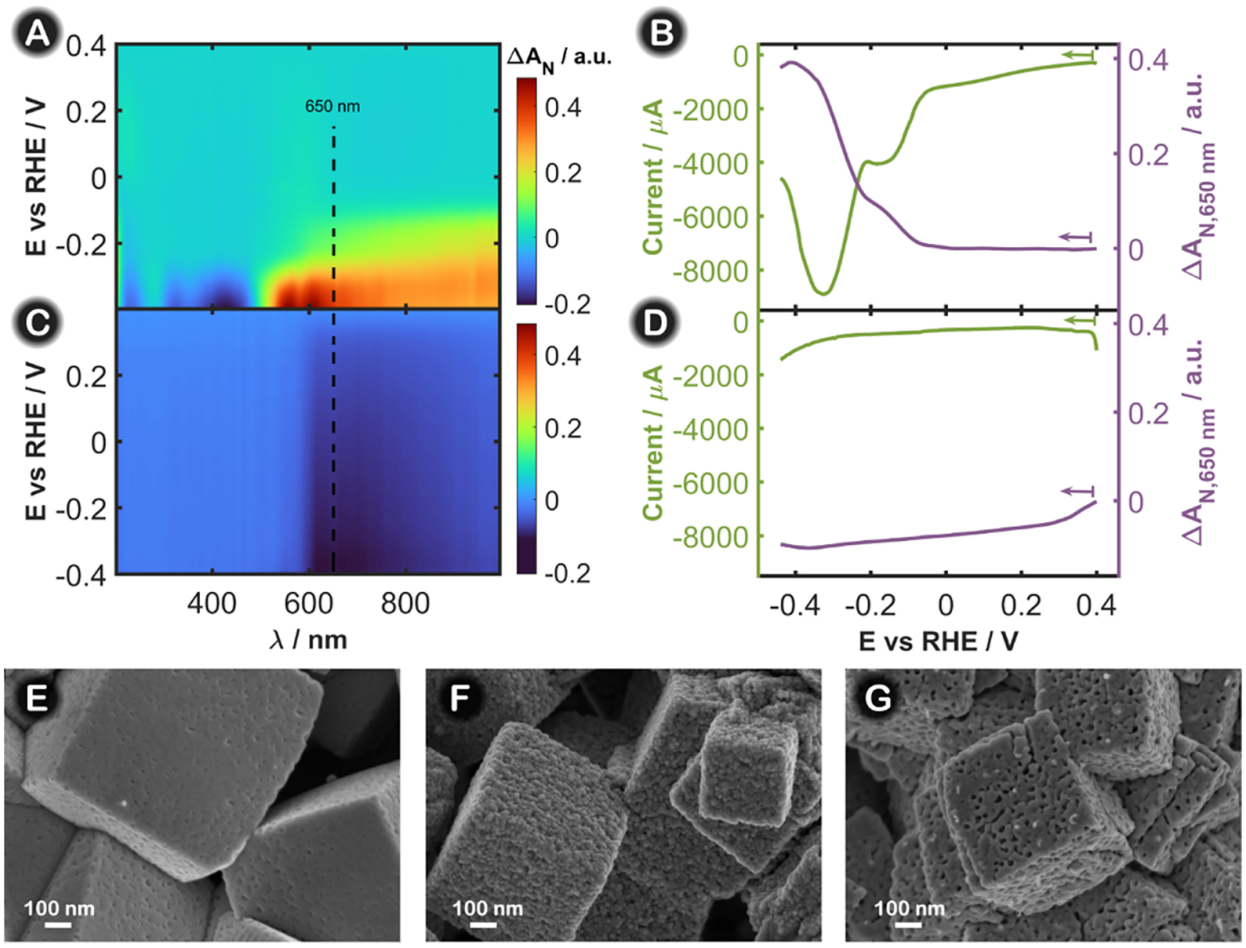
Contour plot showing the spectra evolution in the normal configuration during the first (A) and second (C) LSV of the Cu_2_O-based electrode in a 1 M KOH solution. The LSV (green line) and LVA at 650 nm in the normal configuration (purple line) of the Cu_2_O-based electrode in a solution of 1 M KOH during the first reduction scan (B) and the second reduction scan (D). The scan rate was fixed at 0.02 V s^–1^, and the integration time was 100 ms. SEM images of the Cu working electrode after (E) modification with Cu_2_O nanocubes, (F) the first reduction scan, and (G) the second reduction scan in 1 M KOH.

The overlapping of bands that appear and disappear makes it difficult to define their exact positions, but the isosbestic point indicates the interconversion of species, which is consistent with the reduction of Cu_2_O to Cu^0^. This isosbestic point is placed at 490 nm (2.53 eV), which is around the band gap of Cu_2_O, that takes values between 2.0 and 2.5 eV.^29–31^

The LSV and the corresponding LVA at 650 nm for the first reduction scan (Figure 3B) depict very similar behavior, where the two reduction peaks observed in the voltammogram from 0.00 V downward are associated with two peaks in the evolution of the absorbance at 650 nm with potential. This finding indicates that the changes observed in both signals at the Cu_2_O-based electrode are referred to the same processes. When comparing the LSVs and LVAs at 650 nm for the other reduction scans carried out in 1 M KOH (Figure S5A and D), it is confirmed that the electrode surface reaches a stable behavior in terms of current and absorbance and that an irreversible structural change of the Cu-based catalyst takes place only during the first reduction scan in 1 M KOH, yielding a stable structure in the catalyst enriched in Cu^0^.^18,27^ It should be noted that the evolution of the absorbance from the second reduction cycle onward is not related to any voltammetric feature. In fact, small changes in absorbance could be related to changes in electrode reflectivity, caused by changes in the porous characteristics of the electrode surface promoted by the dynamic dissolution/redeposition of copper nanocubes under electrolytic conditions.^28,33^

After each LSV, the electrode surface morphology was studied by using SEM. Figure 3E and Figure S5E show the SEM image corresponding to the initial state of the freshly prepared Cu_2_O-based electrode, where cubes of 400 nm are observed. Figures 3F and Figure S5F show that after the first reduction scan, there is an increase in the roughness of the nanocubes, which is in line with the first reduction peak in the LSV curve and with the formation of Cu^0^ at the nanostructures.^18,33^ After the second LSV (Figures 3G and Figure S5G), such roughness is less obvious, and the appearance of small porosity features is achieved, which could be due to the leaching process followed by a redeposition of Cu^0^ on the surface of the electrode, as has been reported previously.^33^ The small changes observed in the LVA at 650 nm (Figure 3D, purple line) during the second reduction scan in 1 M KOH could be ascribed to the influence of the potential, as current and absorbance follow the same trend without clear evidence of electron transfer processes, which indicates that the catalyst has reached a stable structure in this potential window. This stability was observed both in 1 M KOH and in the presence of nitrate as the evolution of the spectral features at approximately 650 nm was completely similar in the two experiments (Figure 3D, purple line, and Figure S3C). These small changes of absorbance can be ascribed to the changes in the electrode reflectivity, as has been stated above for the blank experiments.

As in the case of the freshly prepared electrode, characterization of the catalyst after the reduction process in 1 M KOH was performed by using EDX and XRD. Figure S4 shows the results corresponding to the EDX analysis before and after reduction, showing that the amount of oxygen decreases from 8.52 wt % to 2.27 wt % and copper increases from 88.97 wt % to 95.05 wt %. In addition, XRD confirms the reduction of Cu_2_O to Cu^0^ during the electrochemical process. Figure S4A and B show the XRD patterns of the electrode modified with Cu_2_O cubes (blue line) and the cubes after the reduction process in 1 M KOH (orange line). As can be seen, the pristine Cu_2_O cubes present characteristic peaks at 36°, 42°, 62°, and 74°, corresponding to Cu_2_O (ICDS-53322), and finally, the porous cubes after reduction show characteristic peaks of metallic Cu at 43°, 50°, and 75° (JCPDS-04-0836).

In conclusion, the first reduction scan of the catalyst dramatically changes its surface and electronic structure. The reduction of the surface was clearly observed, and the evolution of an absorption band around 650 nm can be ascribed to the generation of Cu^0^, which has been corroborated with ex-situ characterization techniques. Once the catalyst was reduced in the first reduction scan and Cu^0^ was formed, these new nanostructures were stable in the next cycles in the potential window selected, as demonstrated by the small changes in absorbance during the subsequent reduction scans, Figure S5A and D. Moreover, the same morphology of the cubes, obtained by SEM, can be observed after the LSV performed in the presence of nitrate, Figure S6.

### 3.3 2D-UV/vis SEC of NRA at Different Initial Nitrate Concentrations

As previously shown in Section 3.1, experiments in the parallel configuration provide insights into the reduction of nitrate to nitrite and the subsequent reduction of nitrite. This facilitates the deconvolution of the two processes, helping us to understand the apparently straightforward NRA process. To study the effect of the initial nitrate concentration on the electrode process, several experiments were conducted at different nitrate contents. Figure 4A shows the LSVs of four solutions with different initial nitrate concentrations, from 0.025 to 0.1 M, all of them in 1 M KOH. As expected, the cathodic current increases with nitrate concentration. The voltammogram features were quite similar among the four experiments, and only a shift in the potential of the main cathodic peak toward more negative values was observed when increasing nitrate concentration. Such a voltage shift could be related to the difficulty in reducing the reaction intermediates at high nitrate concentrations and/or to the limitation of mass transport under the experimental working conditions. To better understand these observations, the spectroscopic signals in the parallel configuration from nitrite at 350 nm and nitrate at 300 nm were also tracked. As can be distinguished from the LVAs in Figure 4B and C, both nitrate and nitrite follow the behavior described in Section 3.1, with nitrite having a bell-shaped trend with a maximum between +0.20 V and −0.20 V and nitrate having an initial small increase in absorbance, followed by a continuous decrease from −0.10 V downward. It is worth noting that the spectroscopic data were corrected by considering the distance between the optical fibers (optical path length), which was not the same in all of the experiments. The good reproducibility and reliability of the measurements are demonstrated in Figure S7, where three replicates of each nitrate concentration are shown overlapped for the three signals, LSV and LVAs at 300 and 350 nm. Figure S8 shows the absorbance values at 300 nm and −0.30 V and the absorbance values at 350 nm and 0.00 V with respect to the nitrate concentration, demonstrating that the process depends linearly on the initial nitrate concentration and further highlights the quality of the SEC measurements performed due to the good reproducibility achieved. This is an important point because SEC measurements in many studies provide qualitative information but limited reproducibility for quantitative data.

**Figure 4:**
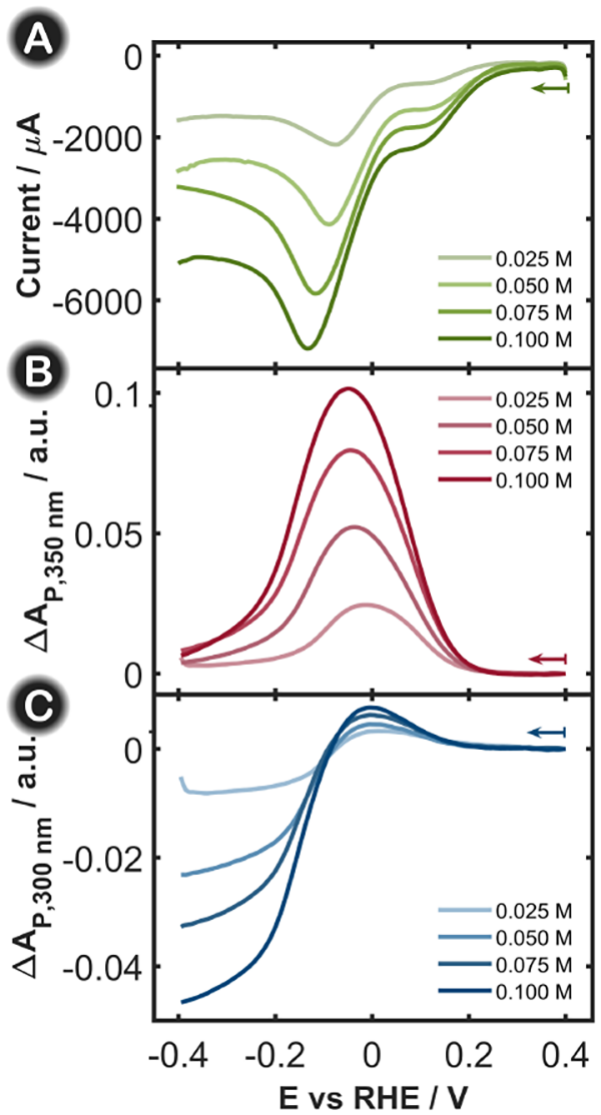
(A) LSVs, (B) LVAs in the parallel configuration at 350 nm, and (C) LVAs in the parallel configuration at 300 nm at four different KNO_3_ concentrations in 1 M KOH. The scan rate was fixed at 0.02 V s^–1^, and the integration time was 10 ms.

As mentioned above (Figure 2C), there is an influence on the absorbance of nitrite in the spectrum of nitrate; therefore, a simple correction of the absorbance spectra was performed to deconvolve the real influence of each anion in the whole measured absorbance in the parallel configuration. Importantly, this correction was made under the assumption that the components detected in the absorption spectra in the parallel configuration were only nitrate and nitrite. Such deconvolution is achieved by determining the molar absorptivity coefficients of nitrate and nitrite at the characteristic wavelengths at which the two anions have the maximum absorption (300 nm for nitrate and 350 nm for nitrite) and the optical pathway of each experiment, which is easily measured in the parallel configuration (Figure S9). The molar absorptivity coefficient of nitrate at 300 nm was 6.34 M^–1^ cm^–1^, whereas that of nitrite at 350 nm was 21.37 M^–1^ cm^–1^. Both agree with the literature.^25,26^

Using this deconvolution protocol, the concentration of each anion was determined and plotted as a function of the potential (Figure 5) to better distinguish its dynamics close to the electrode surface at different initial nitrate concentrations.

**Figure 5:**
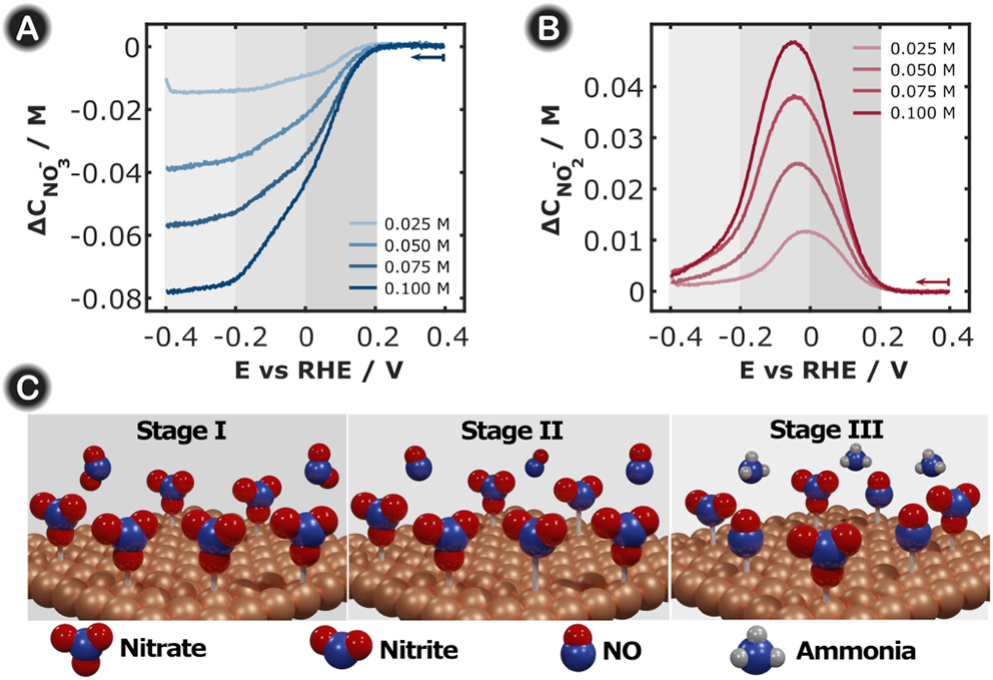
Evolution of the nitrate (A) and nitrite (B) concentrations as a function of the applied potential at different initial nitrate concentrations in 1 M KOH. (C) Schematic illustration of the stages that occur during the nitrate reduction process in 1 M KOH.

From Figure 5, several observations can be drawn. (1) Regardless of the initial nitrate concentration, nitrate reduction occurred at the same cathodic potential, as indicated by the onset potential being practically identical in all experiments, at approximately +0.20 V. As can be seen, nitrite started to be produced at the same potential, at approximately +0.20 V, and more nitrite was generated at a higher initial nitrate concentration. This conclusion is corroborated by the derivative of the absorbance at 350 nm in the parallel configuration (derivative voltabsorptogram, DLVA, Figure S10A) and the potential peak of the positive signal in the DLVA (Figure S10B, blue points), where this positive peak emerges between +0.10 and +0.08 V. (2) Three different regions (Figure 5C) can be distinguished from the concentration profiles of nitrate shown in Figure 5A. The first was between +0.20 and 0.00 V, the second was between 0.00 V and −0.20 V, and the last was from −0.20 V downward. For each region, a different slope can be observed for nitrate consumption in the concentration profiles depicted in Figure 5A. In the first stage, nitrate was almost exclusively converted into nitrite (see model in Figure 5C, stage I), and because of this, the rate of conversion was higher and a higher increase in nitrite concentration was observed, as shown Figure 5B. In the second region, two competitive reduction processes occur simultaneously: nitrate to nitrite reduction and electrochemical nitrite conversion (see model in Figure 5C, stage II). Therefore, the active sites on the Cu-based working electrode should be shared between the nitrate and nitrite. Consequently, the rate of nitrate reduction slowed down, thereby limiting the reduction process. Finally, in the last step, the nitrate concentration remained constant regardless of the application of more cathodic potentials (Figure 5A). Simultaneously, nitrite generation slowed and was consumed in the subsequent reduction process (Figure 5B). In this situation, both nitrate and nitrite were almost completely consumed from the volume sampled by the optical fibers, which implies that the concentration of both anions reached a constant value as a function of potential, reaching a steady state (see the model in Figure 5C, stage III). (3) According to the literature, nitrite reduction on Cu-based electrodes most probably proceeds through the formation of NO,^3,5^ although some other intermediate products as NOOH^–^ have been proposed.^5^ Considering this situation, Figure 5B shows that from 0.00 V to −0.20 V, nitrite is consumed, most probably to form NO on the electrode surface.^34^ DLVAs at 350 nm (Figure S10A) clearly show a negative shift of the nitrate reduction peak, from −0.10 V to −0.16 V as the initial nitrate concentration is increased. This suggests that the concentration of electrogenerated nitrite (which is higher if the initial nitrate concentration increases, Figure 5B) or the formation of NO affects the conversion of nitrite to NO. This shift is clearly observed in the representation of the potential peak of the negative peak from the DLVAs at 350 nm (Figure S10B, red points), where a shift of approximately 70 mV is observed from the lowest to the highest initial nitrate concentration. Consequently, it could be proposed that the product of nitrite reduction or nitrite itself hampers this pathway since it limits the reaction rate.

Therefore, the initial concentration of nitrate plays a key role in the reaction pathway because the higher the initial concentration, the more difficult the reduction of nitrite, indicating that the intermediates of the reaction occupy the catalyst surface sites, hampering the further reduction process.

## 4 Conclusions

2D-UV/vis SEC has been demonstrated to be a powerful technique for following electrocatalytic processes, providing simultaneous information not only about the dynamics of the reactants and products of the NRA process but also about the changes occurring on the catalyst. A new 2D-UV/vis SEC cell has been developed, adapted for the use of optical fibers and compatible with a copper disk electrode. The results demonstrated that the initial catalyst was deeply transformed from the first blank experiment, yielding a catalyst surface with a different electronic structure and composition, which made it active for carrying out the NRA reaction. The dynamics of nitrate and nitrite can be tracked under operating conditions, demonstrating that maximum nitrite production is reached at potentials of approximately 0.00 V. Moreover, NRA is limited by the concentration of nitrate because nitrite formation initially occurs at the same potential for any initial nitrate concentration. However, when the catalyst surface is saturated with the products of the reaction, the reduction of nitrite is limited and facilitated at low concentrations of nitrite on the surface. Further work must be done in the future to obtain spectroscopic information when bubbles are generated on the electrode surface, and SEC devices can also be improved to extract information in the UV region at shorter wavelengths than those observed in this study.

## Supplementary Material



## Notes

Evolution of UV/vis absorption spectra in parallel configuration with potential during a 2D-UV/vis SEC experiment of NRA; comparison of 2D-UV/vis SEC experiments for nitrite reduction reaction and NRA; responses in normal configuration of the 2D-UV/vis SEC experiment of NRA; UV/vis-SEC in normal configuration of the Cu_2_O electrode in 1 M KOH; characterization of the freshly prepared electrode and after pretreatment with 1 M KOH; SEM characterization after reduction in 0.1 M KNO_3_ solution in 1 M KOH; linear sweep voltammetries and linear voltabsorptograms in parallel configuration at different nitrate concentrations; absorbance values in parallel configuration at 300 nm and −0.30 V and at 350 nm and 0.00 V for the different nitrate concentrations; molar absorptivity coefficient assessment for nitrate and nitrite at different wavelengths; derivative voltabsorptograms in parallel configuration and plot of the peak positions at different nitrate concentrations (PDF)

## Abbreviations

NRA: Nitrate reduction to ammonia
HB: Haber–Bosch process
FTIR spectroscopy: Fourier-transform infrared spectroscopy
XAS: X-ray absorption spectroscopy
SERS: Surface-enhanced Raman scattering
FE-SEM: Field-emission scanning electron microscope
XRD: X-ray diffraction
RHE: Reversible hydrogen electrode
SEC: Spectroelectrochemistry
LSV: Linear sweep voltammogram
LVA: Linear voltabsorptogram
DLVA: Derivative linear voltabsorptogram
