# Supplementary material for: Operando UV/vis Absorption Spectroscopy for Studying the Nitrate to Ammonia Conversion on Cu2O‑Based Electrodes
Source: ACS Catal. 2026 Jan 16;16(3):2331–9. doi: 10.1021/acscatal.5c07326 (PMC12888488; doi:10.1021/acscatal.5c07326)
Supplement: Supplementary file 1 [file cs5c07326_si_001.pdf]

Supporting Information

Operando UV/Vis absorption spectroscopy for studying the nitrate to ammonia conversion on Cu_2O-based electrodes

Maria Huidobro[a], Luis Romay[a], Martin Perez-Estebanez[a], Aranzazu Heras[a], Juan. V. Perales-Rondon[b], Alvaro Colina[a]*.*

[a] Department of Chemistry, Universidad de Burgos, Pza. Misael Bañuelos s/n, E-09001, Burgos, Spain.

[b] Hydrogen and Power-to-X Department, Iberian Centre for Research in Energy Storage, Polígono 13, Parcela 31, «El Cuartillo», E-10004, Cáceres, Spain.

* Corresponding authors: juan.perales@ciiae.org, acolina@ubu.es

INDEX

1. Evolution of UV/Vis absorption spectra in parallel configuration with potential during a 2D-UV/Vis SEC experiment of NRA. S-3

2. Comparison of 2D-UV/Vis SEC experiment for nitrite reduction reaction and NRA........... S-4

3. Responses in normal configuration of 2D-UV/Vis SEC experiment of NRA. S-5

4. Characterization of the freshly prepared electrode and after pretreatment with 1M KOH..... S-6

5. UV/Vis-SEC in normal configuration of Cu_2O electrode in 1 M KOH. S-7

6. SEM characterization after reduction in 0.1 M KNO_3 solution in 1 M KOH....... S-8

7. Linear sweep voltammograms and linear voltabsorptograms in parallel configuration at different nitrate concentrations. S-9

8. Absorbance values in parallel configuration at 300 nm and -0.30 V, and at 350 nm and 0.00 V for the different nitrate concentrations....... S-10

9. Molar absorptivity coefficient assessment for nitrate and nitrite at different wavelengths. . S-11

10. Derivative voltabsorptograms in parallel configuration and plot of the peaks position at different nitrate concentrations. S-12

1. Evolution of UV/Vis absorption spectra in parallel configuration with potential during a 2D-UV/Vis SEC experiment of NRA

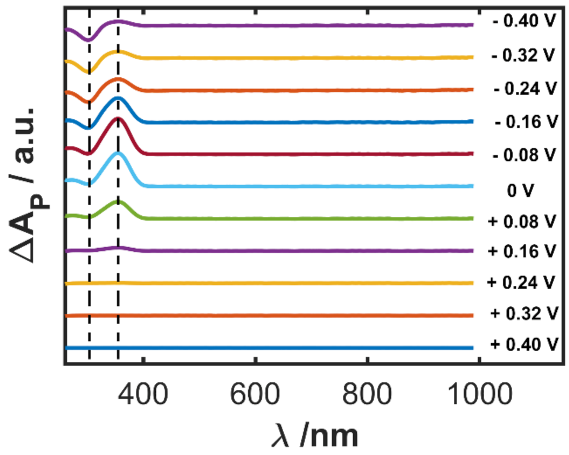

Figure S1. UV/Vis absorption spectra in parallel configuration at different potentials during the reduction scan of a solution 0.1 M KNO_3 in 1 M KOH. The integration time was 10 ms.

2. Comparison of 2D-UV/Vis SEC experiment for nitrite reduction reaction and NRA

The experiments were carried out with nitrite using two different cations (Na and K) to evaluate whether the cation plays a significant role in the observed SEC data.

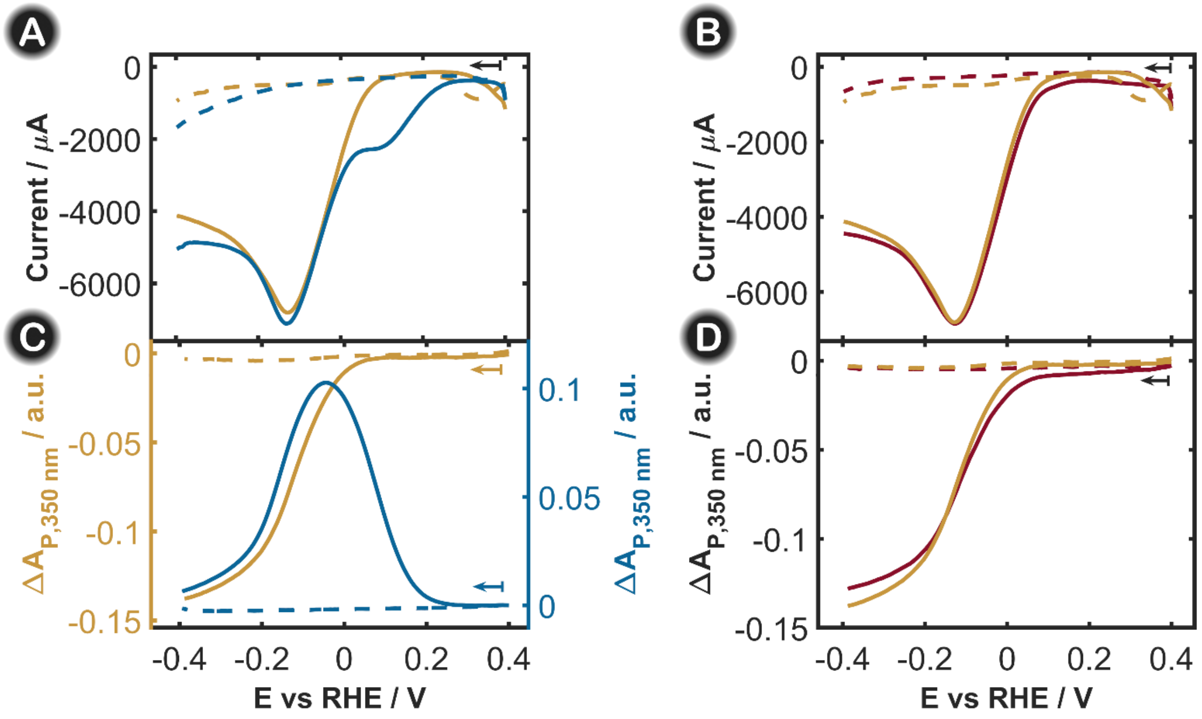

Figure S2. LSV (A) and LVAs (C) in parallel configuration at 350 nm of reduction of 0.1 M KNO_3 (blue line) in 1 M KOH and 0.1 M KNO_2 (yellow line) in 1 M KOH. LSV (B) and LVAs (D) in parallel configuration at 350 nm of reduction of 0.1 M KNO_2 (yellow line) in 1 M KOH and 0.1 M $NaNO_2$ (red line) in 1 M KOH. Experiments were conducted in the absence (dashed line) and presence (solid line) of 0.1 M KNO_3, 0.1 M $NaNO_2$ or 0.1 M KNO_2. Scan rate = 0.02 V s^{-1}. Integration time of 10 ms for the experiment with nitrate and 100 ms for the experiments with nitrite.

3. Responses in normal configuration of 2D-UV/Vis SEC experiment of NRA

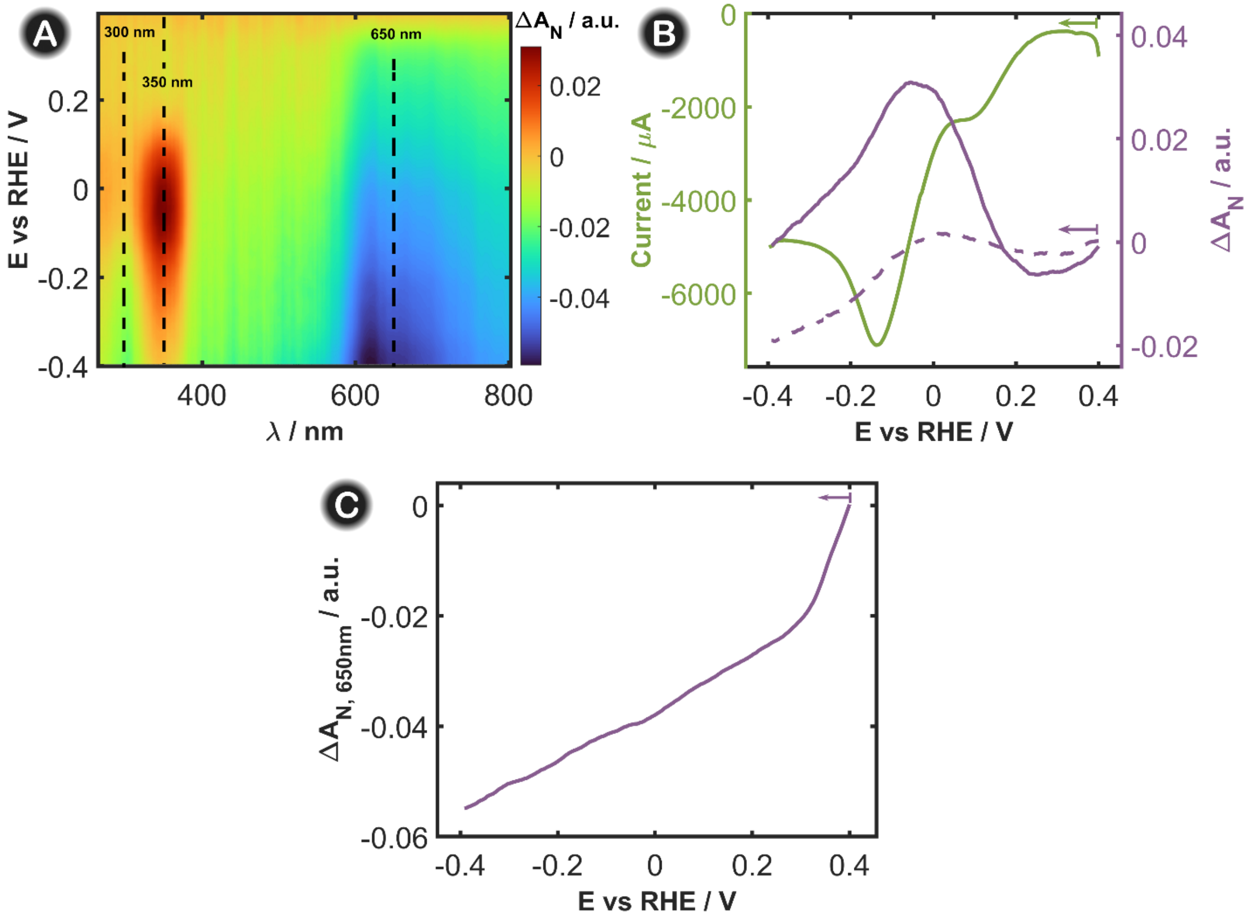

Figure S3. (A) Contour plot showing the evolution of spectra in normal configuration during the LSV of a 0.1 M KNO_3 solution in 1 M KOH. (B) LSV (green solid line), LVA at 350 nm (purple solid line) and LVA at 300 nm (purple dash line) of a solution of 1 M KOH and 0.1 M KNO_3. (C) LVA at 650 nm of a solution of 1 M KOH and 0.1 M KNO_3. Scan rate = 0.02 V s^{-1}, integration time = 100 ms.

4. Characterization of the freshly prepared electrode and after pretreatment with 1M KOH

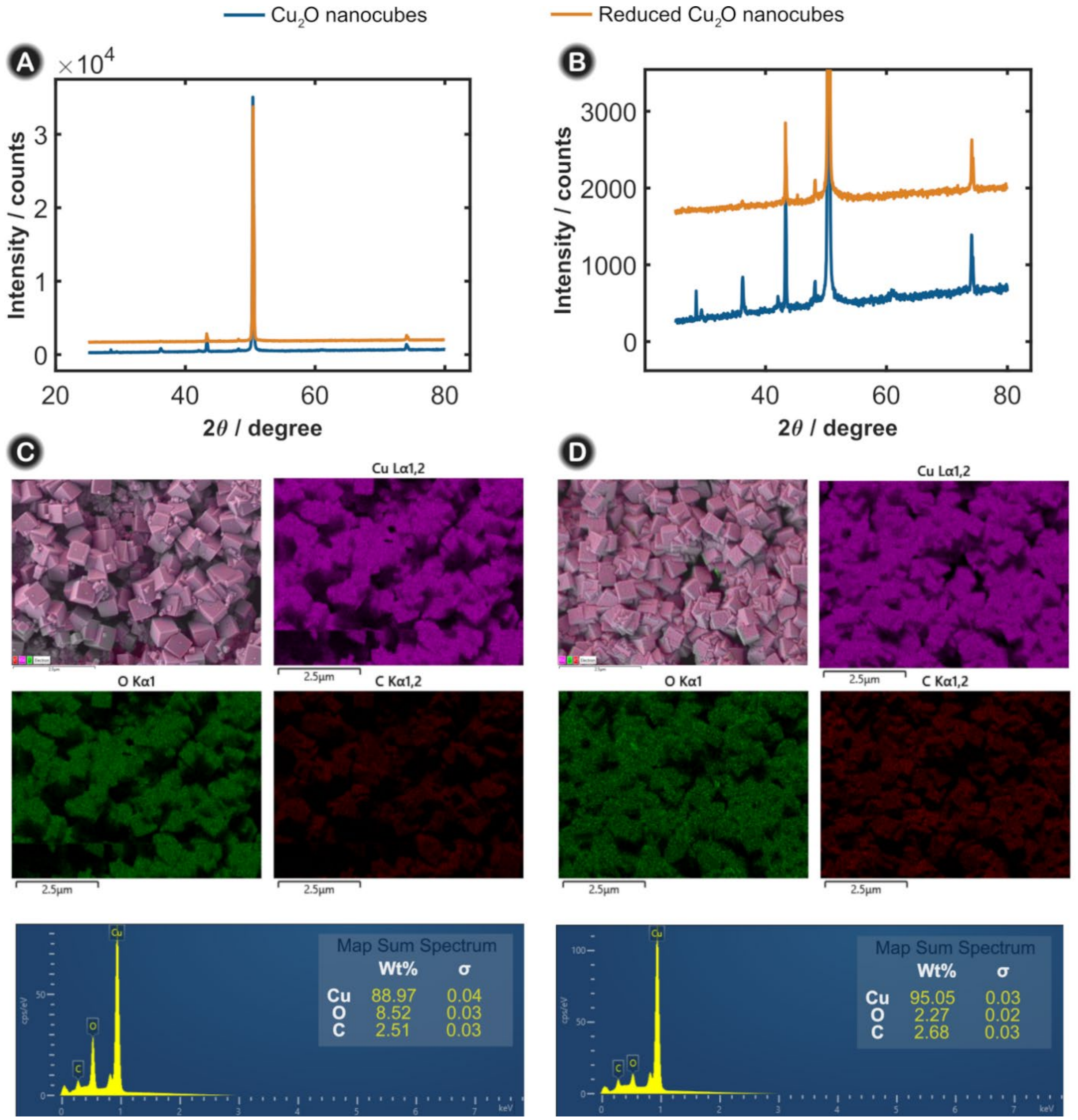

Figure S4. Characterization of the electrode as-prepared (Cu_2O nanocubes) and after pretreatment (reduced Cu_2O nanocubes) in 1 M KOH. (A) XRD spectra of the Cu_2O nanostrucures (blue line) and reduced Cu_2O nanostructures (orange line). (B) Amplification of the XRD spectra in order to better appreciate the peaks related to Cu_2O and Cu. EDX mapping of the (C) Cu_2O catalysts as-prepared and (D) after pretreatment in 1 M KOH solution.

5. UV/Vis-SEC in normal configuration of Cu_2O electrode in 1 M KOH

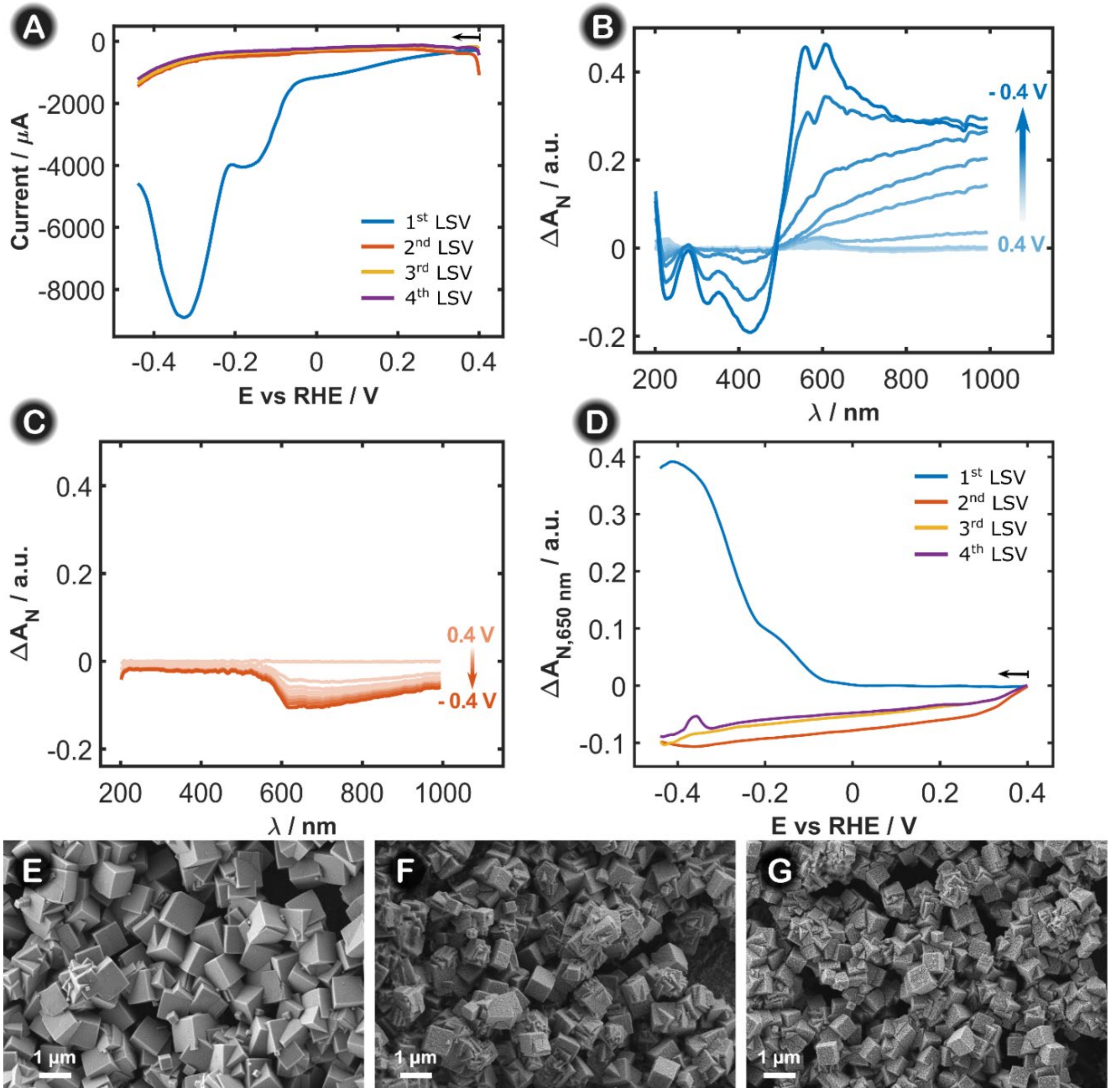

Figure S5. (A) LSVs; evolution of UV/Vis absorption spectra in normal configuration at different potentials during (B) the first reduction scan and (C) the second reduction scan; (D) LVAs at 650 nm. Experiment performed with a Cu_2O-based electrode in 1 M KOH, 4 potential scans. Scan rate = 0.02 V s^{-1}, integration time = 100 ms. SEM images of the Cu working electrode after (E) modification with Cu_2O nanocubes, (F) the first reduction scan, and (G) the second reduction scan in 1 M KOH.

6. SEM characterization after reduction in 0.1 M KNO_3 solution in 1 M KOH

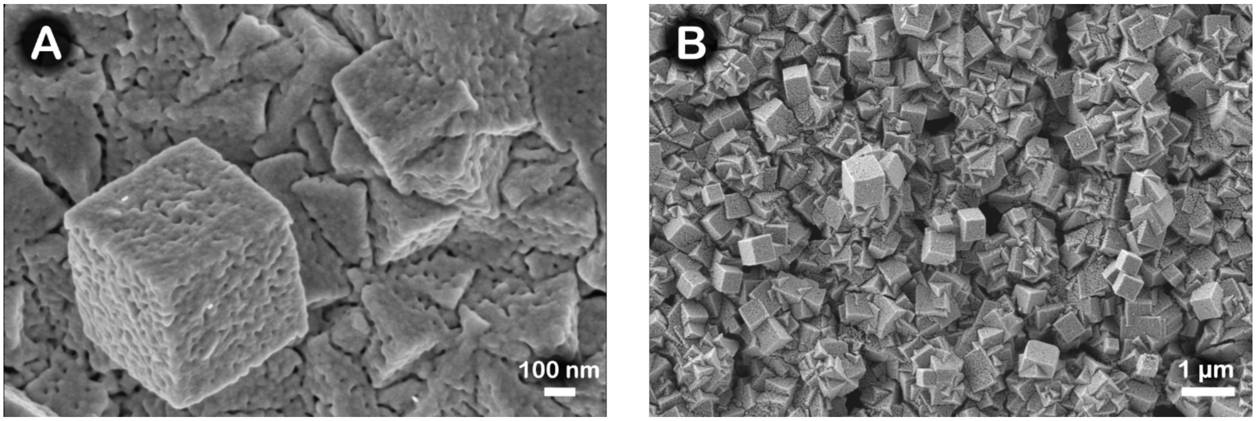

Figure S6. (A) SEM image of the modified Cu working electrode after a reduction scan in a 0.1 M KNO_3 solution in 1 M KOH. (B) SEM image with a lower magnification of the same working electrode.

7. Linear sweep voltammograms and linear voltabsorptograms in parallel configuration at different nitrate concentrations

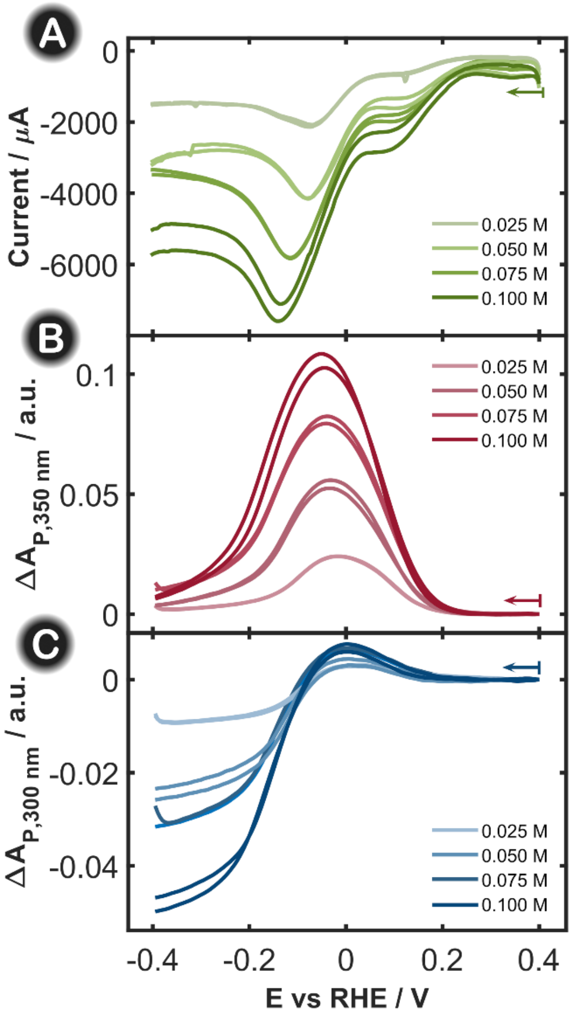

Figure S7. (A) LSVs, (B) LVAs in parallel configuration at 350 nm, and (C) LVAs in parallel configuration at 300 nm at four different KNO_3 concentrations in 1 M KOH. Three replicates of each nitrate concentration are plotted for each signal. Scan rate = 0.02 V s^{-1}, integration time = 10 ms.

8. Absorbance values in parallel configuration at 300 nm and -0.30 V, and at 350 nm and 0.00 V for the different nitrate concentrations

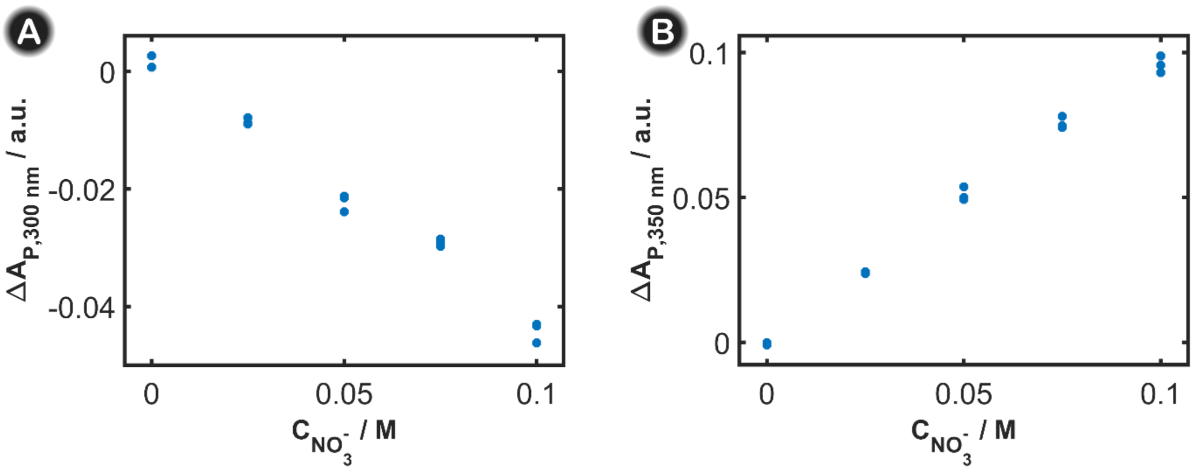

Figure S8. (A) Absorbance in parallel configuration at 300 nm and -0.30 V respect to the nitrate concentration, and (B) absorbance in parallel configuration at 350 nm at 0.00 V respect to the nitrate concentration

9. Molar absorptivity coefficient assessment for nitrate and nitrite at different wavelengths

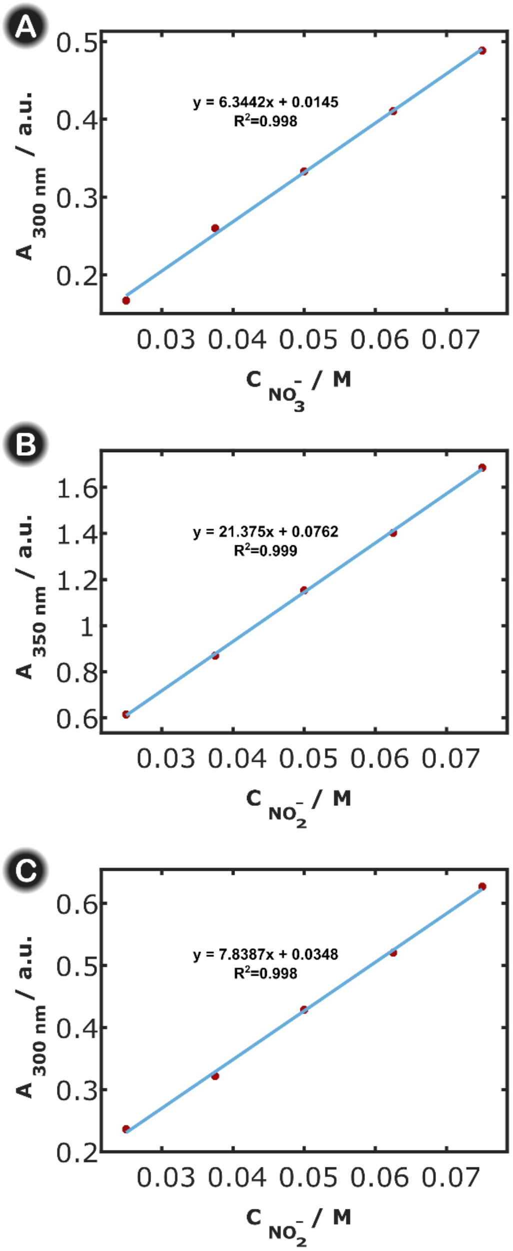

Figure S9. (A) Absorbance of nitrate at 300 nm as a function of nitrate concentration. (B) Absorbance of nitrite at 350 nm as a function of nitrite concentration. (C) Absorbance of nitrite at 300 nm as a function of nitrite concentration. Experiments carried out in a quartz cuvette with an optical path length of 1 cm. The molar absorptivity coefficient for each anion at each wavelength is determined from the Lambert-Beer law by calculating the slope from the corresponding plots.

10. Derivative voltabsorptograms in parallel configuration and plot of the peaks position at different nitrate concentrations

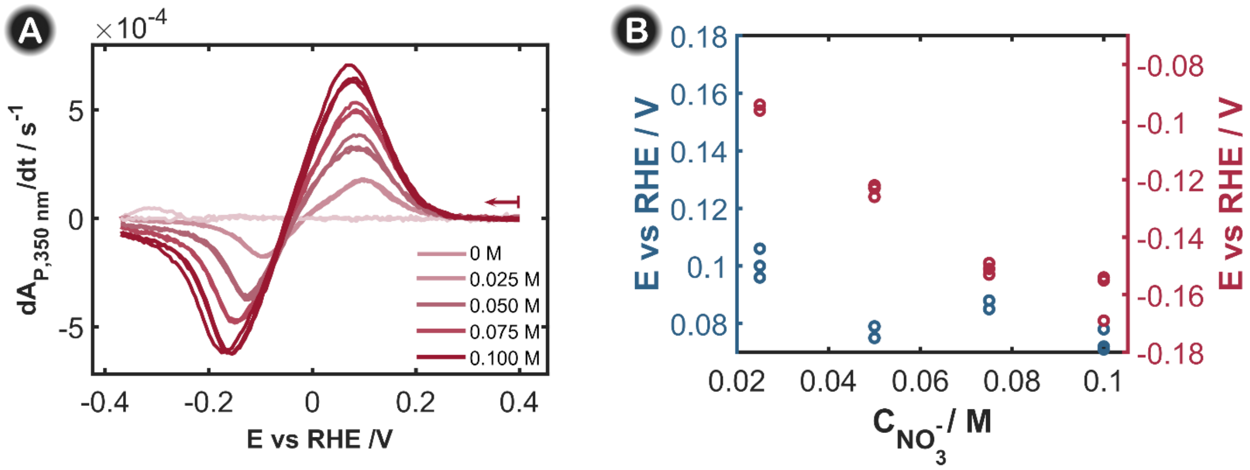

Figure S10. (A) DLVAs at 350 nm in parallel configuration of five solutions with different KNO_3 concentrations in 1 M KOH. (B) Maximum (blue points) and minimum (red points) potential peaks of the DLVAs respect to nitrate concentration.
